# Type A Aortic Dissection With Intramural Hematoma: A Challenging Diagnosis

**DOI:** 10.7759/cureus.33300

**Published:** 2023-01-03

**Authors:** Tayseer Kanaan, Ahmed S Abdelrahman, Jihad Jaber, Amr M Fahmi, Awad Almasalmeh, Soliena Alnakawa, Tala Kanaan

**Affiliations:** 1 Cardiology, Hamad Medical Corporation, Doha, QAT; 2 Pharmacy, Hamad Medical Corporation, Doha, QAT; 3 Radiology, Primary Health Care Corporation, Doha, QAT; 4 Internal Medicine, Hamad Medical Corporation, Doha, QAT

**Keywords:** intramural hematoma, type a aortic dissection, aortic dissection, acute aortic syndrome, chest pain

## Abstract

Intramural hematoma (IMH) is considered a part of acute aortic syndromes (AAS), a group of life-threatening aortic diseases with a similar presentation that appears to have different clinical manifestations and pathological and survival characteristics. AAS comprises three major entities, namely, aortic dissection (AD), IMH, and PAU. IMH-like classic AD is classified using Stanford and DeBakey classification systems to indicate the aortic area involved.

Early diagnosis and treatment of AAS are crucial for survival; however, diagnosis of IMH may be delayed and challenging due to atypical presentation, investigation findings, and case progression. In this report, we describe a case of delayed and challenging diagnosis of a Stanford type A IMH that was managed surgically with a good outcome.

## Introduction

Acute aortic syndrome (AAS) describes a group of life-threatening aortic diseases (ADs). It comprises three major entities, namely, AD, intramural hematoma (IMH), and penetrating aortic ulcer (PAU) [1]. The aortic wall consists of three layers (intima, media, adventitia). AD compromises the majority of AAS and is characterized by a separation within the medial layer of the aortic wall caused by an intimal tear. This results in the creation of a true lumen and a false lumen which causes the blood to flow in either direction, i.e., antegrade or retrograde [2]. IMH is a collection of blood that results from the rupture of the vasa vasorum into the media layer of the aorta without an intimal tear or flap formation [3,4]. Hypertension and atherosclerosis are the most common risk factors in the development of IMH, PAU, and AD. Other risk factors include prior cardiac surgery, aortic aneurysm, connective tissue disorder (e.g., Marfan syndrome), bicuspid aortic valve, and prior aortic surgery [5]. The incidence of IMH ranges from 5% to 25% of AAS. Compared to acute AD, IMH is more prevalent in elderly patients [6].

IMH is classified using Stanford type A or B and DeBakey classification systems to indicate the aortic area involved. Most patients (50-85%) with IMH have Stanford type B. Clinical manifestations are similar in AAS patients, i.e., chest pain such as classic AD aortic pain. Rarely, patients may present with syncope, anterior spinal pain syndrome, hoarseness, or acute renal insufficiency [7]. Conclusive diagnosis of AAS and its complications is obtained by the use of noninvasive imaging modalities, such as computed tomography angiography (CTA), magnetic resonance imaging (MRI), and echocardiography (both transthoracic and transesophageal) [8].

As CTA exposes patients to radiation and large doses of intravenous contrast, and transesophageal echocardiogram (TEE) and MRI may not be available or performed at the appropriate time, there are ongoing attempts to find simple and available methods that can help clinicians avoid misdiagnosis and overtesting of AAS. For that aim, studies have shown the importance of D-dimer and Aortic Dissection Detection Risk Score (ADD-RS) in the diagnosis of AAS. D-dimer is a useful screening tool to identify patients who do not have acute aortic dissection using a cutoff of 500 ng/mL. A level below this value is highly predictive of excluding dissection [9]. ADD-RS is based on the presence of one or more of the following: (1) high-risk conditions such as Marfan syndrome, family history of AS, presence of an aortic aneurysm, etc; (2) pain in the chest, back, or abdomen described as abrupt and of severe intensity (aortic pain); (3) physical examination findings of perfusion deficit, such as pulse deficit, systolic blood pressure difference, or focal neurologic deficit. The presence of at least one marker within each of these groups is given a score of 1 with a maximum cumulative score of 3 if all three are present. High ADD-RS effectively stratifies the risk for acute aortic dissection [10]. The addition of D-dimer to ADD-RS may improve diagnostic performance compared with each of these when used alone for ruling out acute aortic dissection [11,12]. A multicenter study in which AAS was considered a possibility showed that the combination of ADD-RS (0-1) and negative D-dimer (<500 mg/mL) effectively rules out AAS with a failure rate of less than one in 300 patients [13]. Based on these results, about 60% of patients with a low probability of AAS might be spared from unnecessary conclusive vascular imaging. This is a novel clinical strategy for evaluating patients with the potential of AAS but still requires external validation before implementation into clinical practice.

The incidence of initial misdiagnosis of AAS is up to 40% and may be more common when the ascending aorta is involved [14,15]. Periaortic hematoma and hemorrhagic pericardial effusion are the major complications of AAS that occur more frequently in IMH than in acute AD and can be fatal unless treated on an emergency basis [4,16]. Progression of IMH is variable; it may be reabsorbed without any intervention, or it may progress to classic aortic dissection, with outward aortic rupture observed in 15-20% of patients [7]. In general, early surgery is recommended for patients with type A IMH, whereas many patients with type B IMH can be managed conservatively in the absence of complications with the resolution of the aortic abnormality over time [7,17].

Patients with AAS can present with a non-specific complaint or progress atypically which makes early diagnosis difficult. This report presents a case of delayed and challenging diagnosis of Stanford type A IMH that progressed to classic aortic dissection which was managed successfully by surgery to repair the aorta.

## Case presentation

We present the case of a 47-year-old male Sri Lankan patient with a history of hypertension and type 2 diabetes mellitus on regular medications. The patient also had a history of right kidney hydronephrosis three years before presentation, which completely resolved during follow-up. The patient had no other cardiac risk factors, no cardiac history, no surgical history, and no family history of cardiac disease or sudden cardiac death. Apart from medication for hypertension and diabetes, the patient denied using any other medications.

The patient presented to the emergency department with sudden onset of central chest pain that started two hours before the presentation. The pain was severe, continuous, and radiated to the back. There were no associated symptoms and had no other complaints.

The patient’s vital signs and measurements were as follows: height: 165 cm, weight: 81 kg: temperature: 36.6°C (oral), blood pressure: right arm 105/62 mmHg and left arm 110/67 mmHg, heart rate: 73 beats/minute, respiratory rate: 18 breaths/minute, oxygen saturation: 100% on room air.

On presentation, the patient was conscious and oriented and was still complaining of chest pain despite receiving morphine (IV 5 mg). Jugular venous pressure was not raised. Pulse was synchronized and equal in four limbs (no pulse deficit). On heart examination, S1-S2 was normal and regular, without murmurs or additional sounds. The chest was clear. On examination of the abdomen, it was soft and non-tender, without scars, organomegaly, and masses. On examination of the central nervous system, the patient was alert, with no focal neurological findings. Lower limbs: There was no pitting edema in the lower limbs, with no signs of deep vein thrombosis.

**Figure 1:**
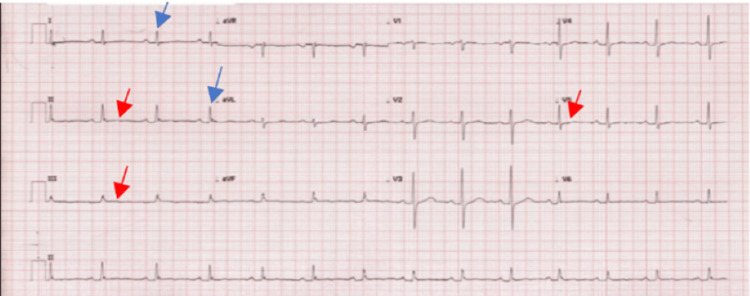
Electrocardiogram showing sinus rhythm with low voltage and non-specific ST-T changes. Blue arrows: low voltage. Red arrows: non-specific ST-T changes.

**Figure 2:**
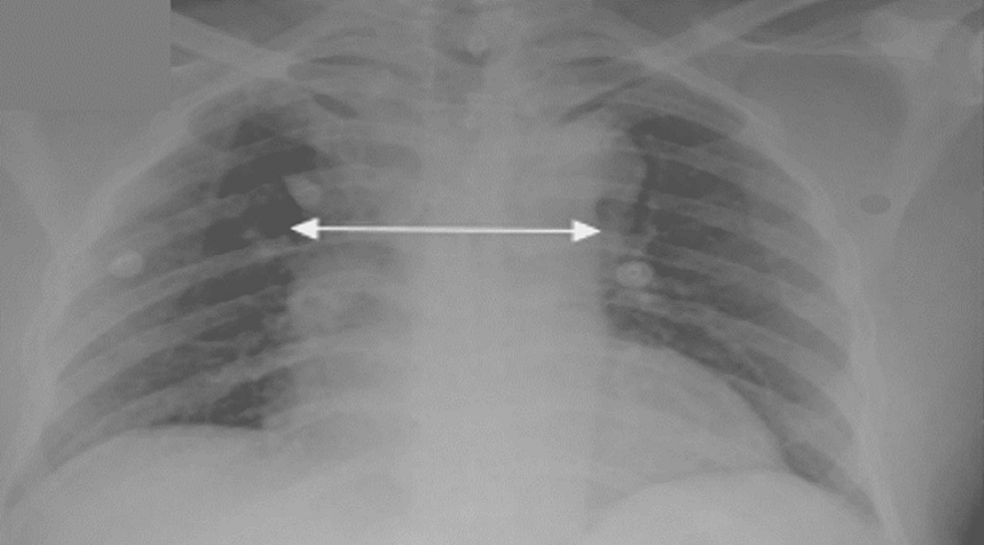
Chest X-ray in the semi-sitting position showing the widening mediastinum (arrow).

The electrocardiogram (ECG) showed sinus rhythm and low voltage with non-specific ST-T changes (Figure 1). Right and posterior leads were normal. A chest X-ray in a semi-sitting position showed mediastinal widening (Figure 2).

The patient’s troponin T was <10 ng/L, white blood cell (WBC) count was 9,400/µL, hemoglobin was 12.6 g/dL, platelet count was 270,000/µL, and international normalized ratio (INR) was 1.1. Thyroid, renal, and liver functions were normal. D-dimer was 0.30 mg/L. Other lab tests were unremarkable.

**Figure 3:**
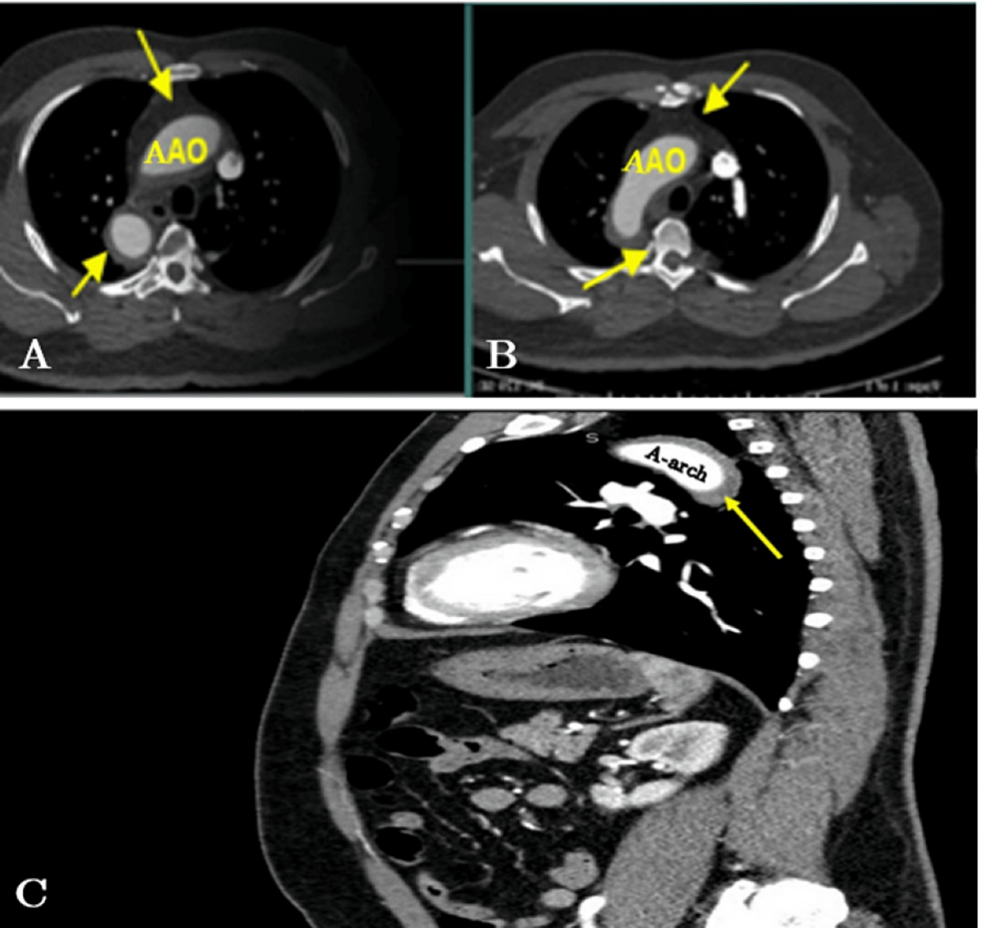
CT of the aorta with no radiological evidence of dissection or aneurysmal dilation (atherosclerotic changes). (A) Ascending aorta: intimal thickening of the aorta wall (yellow arrows). (B) Ascending aorta: intimal thickening of the aorta wall (yellow arrows). (C) Aortic arch: intimal thickening of the aorta wall (yellow arrow). Yellow arrows in A and B show the ascending aorta, while the arrow in C shows the aortic arch. CTA = computed tomography; AAO = ascending aorta; A-arch = aortic arch

Due to continuous chest pain and mediastinal widening on chest X-ray, CTA with contrast was requested to rule out AD. CTA showed thickening of the intimal wall with atherosclerotic calcification without any evidence of dissection or aneurysmal dilation (Figure 3).

**Figure 4:**
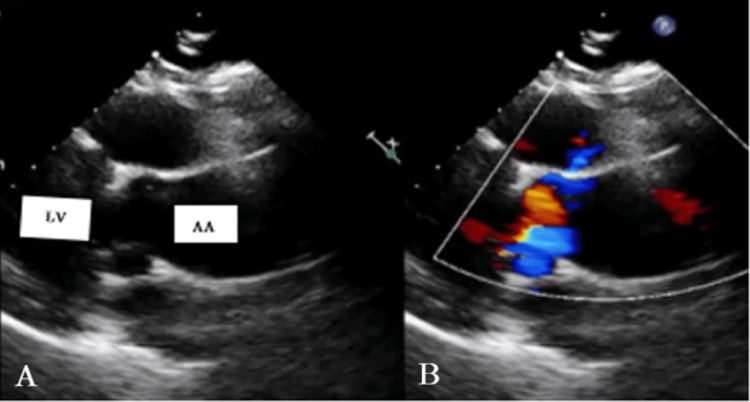
(A) Parasternal long-axis view showing the ascending aorta and the left ventricle. (B) Parasternal long-axis view with color. AA = ascending aorta; LV = left ventricle

Transthoracic echocardiography showed an ejection fraction (EF) of 68%, which indicated a normal study (Figure 4).

The patient was admitted under cardiology care as a case of chest pain for evaluation with a possible diagnosis of acute coronary syndrome. Anti-ischemic treatment was initiated (bisoprolol 2.5 mg daily, enoxaparin 80 mg BID, atorvastatin 80 mg daily, aspirin 100 mg daily, and clopidogrel 75 mg daily).

On the evening of the admission day (after 15 hours of admission), the patient was still complaining of chest pain, and he developed severe epigastric pain and vomiting. At that time, he was vitally stable. No new ECG changes and no elevation in cardiac enzymes were noted. Physical examination showed no new findings. Troponin T: four sets negative), WBC count 9,400 to 12,200/µL, C-reactive protein (CRP) 65 mg/L, lipase 14 U/L (normal), D-dimer 0.30 mg/L, and procalcitonin 0.32 ng/mL.

Due to severe epigastric pain and vomiting, the patient was evaluated by the general surgeon who advised to hold antiplatelet and anticoagulants as the patient possibly needed surgery. Moreover, the patient stayed on pain management medication (paracetamol 1,000 mg oral TID PRN for pain), oral antidiabetics, antihypertensives, and antibiotics until he was transferred to CT surgery.

**Figure 5:**
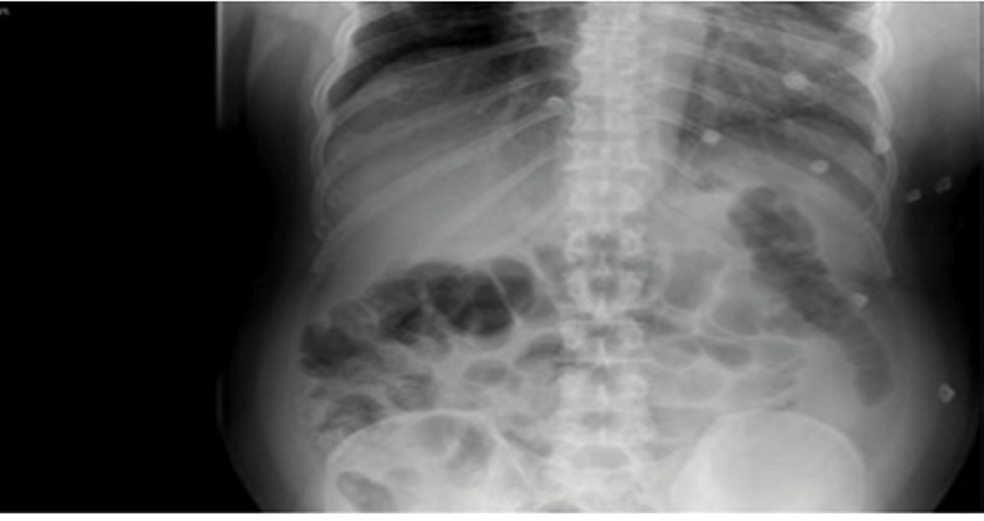
Abdominal X-ray.

**Figure 6:**
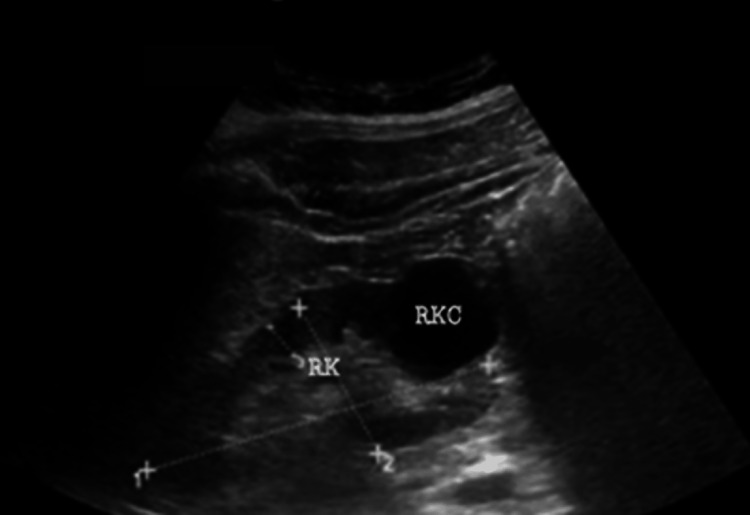
Abdominal ultrasound. RK = right kidney; RKC = right kidney cyst

The general surgeon requested abdominal ultrasound and X-ray. The abdominal X-ray was normal. Abdominal ultrasound was also normal except for a right cortical kidney (Figures 5, 6).

On the second day of admission, the patient was still complaining of chest pain, severe epigastric pain, and vomiting, and he developed a low-grade fever. He appeared uncomfortable and vitally stable, with no new ECG changes. On examination, no new findings were noted. TroponinT five sets were negative.

The general surgery and urology teams evaluated the patient (who was cleared from their side). He was also evaluated by the internal medicine team because of abdominal pain, vomiting, and fever. They suggested an infection workup, blood culture aerobic and anaerobic, procalcitonin, and urinalysis to rule out urinary tract infection.

On the third, fourth, and fifth day of admission, the patient was still complaining of epigastric pain, vomiting, and high-grade fever (39°C). On examination, there were no new significant findings. On ECG, there were no new changes. Troponin T five sets were negative. There was a significant elevation of inflammatory markers. WBC count increased from 9,400 to 12,200 to 13,600/µL. CRP from 65 to 359 mg/L. Blood culture showed no growth. At that time, the patient was re-evaluated by the internal medicine team which suggested starting ceftriaxone 2 g IV OD and to follow-up with a urine dipstick, urine culture, and blood culture.

On the sixth day of admission, the patient had the same complaints, with no new findings. Because of the fever and elevation of inflammatory markers, the infectious disease team was consulted, and they suggested changing ceftriaxone to piperacillin/tazobactam. They also suggested repeating septic workup, and abdomen/pelvic CT to rule out any collection.

**Figure 7:**
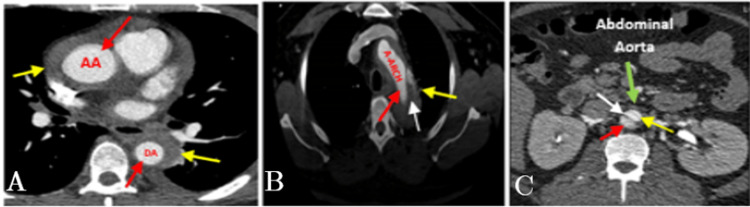
CT of the aorta demonstrates Stanford type A aortic dissection. (A) Ascending aorta. (B) A-arch: the red arrow showing the true lumen, the yellow arrow showing the false lumen, and the white arrow showing the flap. (C) The blue arrow showing the abdominal aorta, the red arrow showing the true lumen, and the yellow arrow showing the false lumen. CTA = computed tomography; DA = descending aorta; AA = ascending aorta; A-arch = aortic arch

CT of the abdomen and pelvis was done which showed that the patient had an abdominal AD; hence, CT with contrast for the whole aorta was done which showed Stanford type A AD (Figure 7).

Once the patient was diagnosed with AAD, he was promptly transferred to a CT surgeon where he underwent cardiothoracic surgery for aortic repair. According to the CT surgeon’s report, the patient was found to have type A AD caused by intramural hematoma that extended from the aortic root and involved the ascending arch of the aorta and beyond. No intimal tear was seen. The ascending aorta was excised and replaced by an interposition graft. The patient had a smooth hospital course and was discharged in a good condition. The patient underwent regular follow-ups in the cardiothoracic outpatient clinic. A follow-up CT for the whole aorta was done twice after surgery (six months and 18 months), which showed no evidence of a complicated graft or recent AD.

## Discussion

IMH is a new entity of AD that ranges from 5-25% of AAS. It is more prevalent in the elderly and women [6]. The final diagnosis can be made by non-invasive imaging modalities, such as echocardiography, CTA, and MRI [8]. A combination of D-dimer and ADD-RS is a simple method that can help confirm or rule out the diagnosis of AAS [13].

However, the diagnosis of AAS is frequently mistaken for other etiologies that cause chest pain, with the most common being acute coronary syndrome. The incidence of initial misdiagnosis is up to 40% and may be more common when the ascending aorta is involved [14,15].

In our case, four factors contributed to the difficulty and delay of the diagnosis. The first CT scan of the whole aorta (which was done on the first day of admission) did not show any evidence of AAD, rather there was a mild thickness in the wall of the aorta which was considered by the radiology team to be atherosclerotic changes. Transthoracic echocardiography was negative for AAD. There was a low probability of AAD according because ADD-RS was 1 and D-dimer was 300 ng/mL cut-off (<500 ng/mL). There was an abnormal progression of the patient after a few hours of admission as he had a fever and an elevation in the inflammatory markers as follows: temperature 39°C, WBC count 9,400/µL to 12,200/µL to 136,00/µL; and CRP 65 mg/L to 359 mg/L.

**Figure 8:**
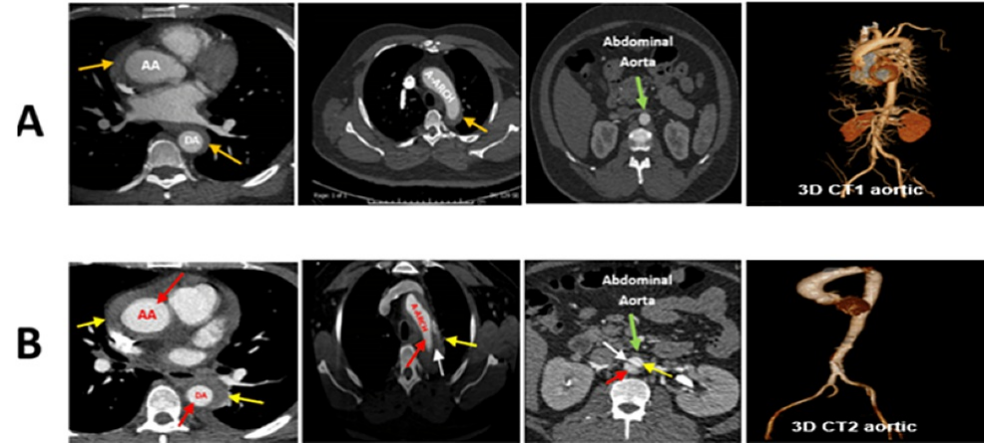
Comparison between the two CT scans of the whole aorta. (A) Done upon admission and showed no evidence of AAD. (B) Done on the sixth day of admission and showed AAD. (A) First CT and 3D CT of the whole aorta upon admission: orange arrows: the thickness of the aorta wall, without evidence of dissection (atherosclerotic changes). (B) Second CT and 3D CT of the whole aorta on the sixth day of admission demonstrates Stanford type A aortic dissection; red arrows: true lumen, yellow arrows: false lumen, white arrows: flap, and green arrows: abdominal aorta AAD = abdominal aortic dissection; AA = ascending aorta; DA = descending aorta, A-arch = aorta arch

The diagnosis of AD was confirmed on the sixth day based on the results of the second CT scan (done on the sixth day of admission) that showed findings of classic type A AD. The diagnosis of AAS was not present at the time of admission based on the first CT of the whole aorta with contrast (done upon admission) that showed only thickness of the aortic wall which was considered by the radiology team as atherosclerotic changes, without evidence of dissection or aneurysmal dilation (Figure 8A).

The possibility of IMH was diagnosed retrospectively on the sixth day of admission based on the result of the second CTA, as it was believed that the classical signs of AAD of the second CTA probably resulted from the progression of IMH which was excluded upon admission because of the previously mentioned reasons. Second, the CT report confirmed the presence of extensive type A AD caused by IMH that extended from the aortic root to involve the ascending aorta and the arch of the aorta and beyond. No intimal tear was noted.

When the diagnosis of AD was confirmed, the CT surgeon was informed, and the patient promptly underwent cardiothoracic surgery for aortic repair.

Our patient also developed high-grade fever and significant elevation of inflammatory markers which may occur in patients with AAD. The presence of these features, especially CRP elevation, is considered a sign of poor prognosis and is associated with high morbidity and mortality [18,19].

As noted above, there were several challenges in this case (imaging and laboratory test results in addition to case progression) that contributed to the delay in the diagnosis of AAS. We believe that these findings have not been discussed before in the literature which adds to the uniqueness of this case report.

## Conclusions

AAS describes a group of life-threatening conditions that should be a part of the differential diagnosis of patients presenting with chest pain because early diagnosis and treatment of AAS are crucial for patient survival. Because the diagnosis of AD can be misleading, normal imaging findings should be reviewed if there is a high suspicion of AAS. Repeating imaging or using different modalities may be required in certain cases. Unfortunately, some findings (clinical, laboratory, or imaging) may misguide clinicians from the right diagnosis. Therefore, it is important to correlate investigation results with clinical findings, especially in the presence of persistent symptoms. Finally, correlating investigation results with clinical findings and knowing the uncommon and atypical presentations of the disease can save effort and time to reach the diagnosis.
